# Anti-leukemia activity of NSC-743380 in SULT1A1-expressing acute myeloid leukemia cells is associated with inhibitions of cFLIP expression and PI3K/AKT/mTOR activities

**DOI:** 10.18632/oncotarget.22235

**Published:** 2017-11-01

**Authors:** Xiao Huang, Mengru Cao, Shuhong Wu, Li Wang, Jing Hu, Reza J. Mehran, Jack A. Roth, Stephen G. Swisher, Rui-Yu Wang, Hagop M. Kantarjian, Michael Andreeff, Xiaoping Sun, Bingliang Fang

**Affiliations:** ^1^ Department of Thoracic and Cardiovascular Surgery, The University of Texas MD Anderson Cancer Center, Houston, Texas 77030, USA; ^2^ Department of Leukemia, The University of Texas MD Anderson Cancer Center, Houston, Texas 77030, USA; ^3^ Department of Laboratory Medicine, The University of Texas MD Anderson Cancer Center, Houston, Texas 77030, USA; ^4^ Department of Traditional Chinese Medicine, Beijing Friendship Hospital, Capital Medical University, Beijing 100050, China

**Keywords:** cancer, drug development, biomarker, sulfotransferase, SULT1A1

## Abstract

Our recent study showed that acute myeloid leukemia (AML) cells expressing SULT1A1 are highly sensitive to NSC-743380, a small molecule that inhibits STAT3 activity and induces SULT1A1-dependent apoptosis of various cancer cell lines. In this study, we characterized the molecular mechanisms of NSC-743380–mediated anti-leukemia activity in AML cell lines and antileukemia activity of NSC-743380 in patient-derived primary leukemia cells from AML patients. Our results showed that treatment with NSC-743380 triggered robust apoptosis in SULT1A1-positive AML cells. Treatment with NSC-743380 did not increase intracellular reactive oxygen species or change of STAT3 activity in AML cells, but did dramatically and rapidly decrease cFLIP expression. Proteomic analysis with reverse phase protein microarray revealed that treatment of U937 and THP-1 AML cells with NSC-743380 led to drastic and time-dependent suppression of phosphorylation of several key nodes in the PI3K/AKT/mTOR pathway, including AKT and mTOR. Moreover, primary AML cells expressed SULT1A1 were highly sensitive to treatment with NSC-743380, which was not affected by co-culture with bone marrow mesenchymal stem cells. Thus, our results provide proof-of-concept evidence that AML cells expressing SULT1A1 can be targeted by small molecules that induce apoptosis through inhibiting the expression or activities of multiple targets.

## INTRODUCTION

Acute myeloid leukemia (AML) is a group of malignant diseases of the hematopoietic system with remarkable heterogeneity in their cytogenetic and genomic alterations. With an estimated annual incidence of 21,380 and with 10,590 associated deaths annually, AML accounts for the largest number of annual deaths due to leukemia in the United States [1]. Patterns of cytogenetic aberrations and gene mutations have been used to divide AML patients into subgroups that are associated with clinical phenotype and outcome [2, 3], and such subgroups may guide selection of patients for novel pathway targeted therapies. Although classical induction therapy with cytarabine and daunorubicin has been used to treat AML over the past 40 years [4, 5], recent studies have demonstrated that certain innovative therapeutic approaches can dramatically improve the clinical outcomes of specific subsets of AML patients [6]. For example, treatment of acute promyelocytic leukemia with all-trans retinoic acid and arsenic trioxide improved survival rate from 75% to 95% [7]. AML with aberrant DNA methylation resulting from TET2 or IDH2 mutations is susceptible to 5-azacytidine or to IDH2 inhibitor in experimental models [8], demonstrating the feasibility of improving clinical outcomes of AML patients using novel targeted agents.

Through synthetic lethality–based chemical library screening, we previously identified a small molecule, called oncrasin-1, that selectively induced apoptosis in KRAS-mutant cancer cells [9]. Optimization of this lead compound by syntheses and evaluations of analogues through a collaboration with the Developmental Therapeutics Program at the National Cancer Institute led to the development of NSC-743380 [10], which selectively killed a subgroup of cancer cell lines derived from lung, breast, ovarian, colon, and renal cancers [11, 12]. Mechanistic characterization revealed that NSC-743380 and its analogues induced apoptosis [11, 12], inhibited phosphorylation of the C-terminal domain of RNA polymerase II [10, 13], induced sustained JNK activation by inhibiting its dephosphorylation [14], induced intracellular reactive oxygen species (ROS) accumulation [15, 16], inhibited STAT3 phosphorylation, and suppressed cyclin D1 expression [11], suggesting that NSC-743380 modulates multiple cancer-related targets. *in vivo* studies showed that the intravenous or intraperitoneal administration of NSC-743380 caused complete tumor regression or significant growth suppression in xenograft tumor models derived from renal cancer cell A498 and lung cancer cell H157 [11, 12]. Upon correlation analysis of the anti-cancer activity of NSC-743380 and the gene expression levels in NCI-60 cell lines as well as functional characterization of the top genes associated with NSC-743380–mediated anticancer activity, we recently reported that expression of the sulfotransferase SULT1A1, a biotransformation enzyme that bioactivates a number of pro-carcinogens [17], is causally associated with the selective anti-cancer activity of NSC-743380 [18]. Moreover, we found that a subset of leukemia cell lines, mostly AML cells, express SULT1A1 and are highly sensitive to NSC-743380. Nevertheless, whether NSC-743380 has similar mechanisms of action in AML cells as that observed in solid cancer cells is unknown. The purpose of this study was to characterize molecular alterations induced by NSC-743380 in NSC-743380–susceptible AML cell lines and measure the *in vitro* activity of NSC-743380 against primary leukemia cell samples. Our results showed that NSC-743380 induces robust apoptosis, abrogates the expression of cFLIP, and inhibits the activity of the PI3K/AKT/mTOR pathway in AML cells and induces robust apoptosis in primary AML samples that express SULT1A1.

## RESULTS

### NSC-743380 induces robust apoptosis in SULT1A1-expressing AML cells

**Figure 1:**
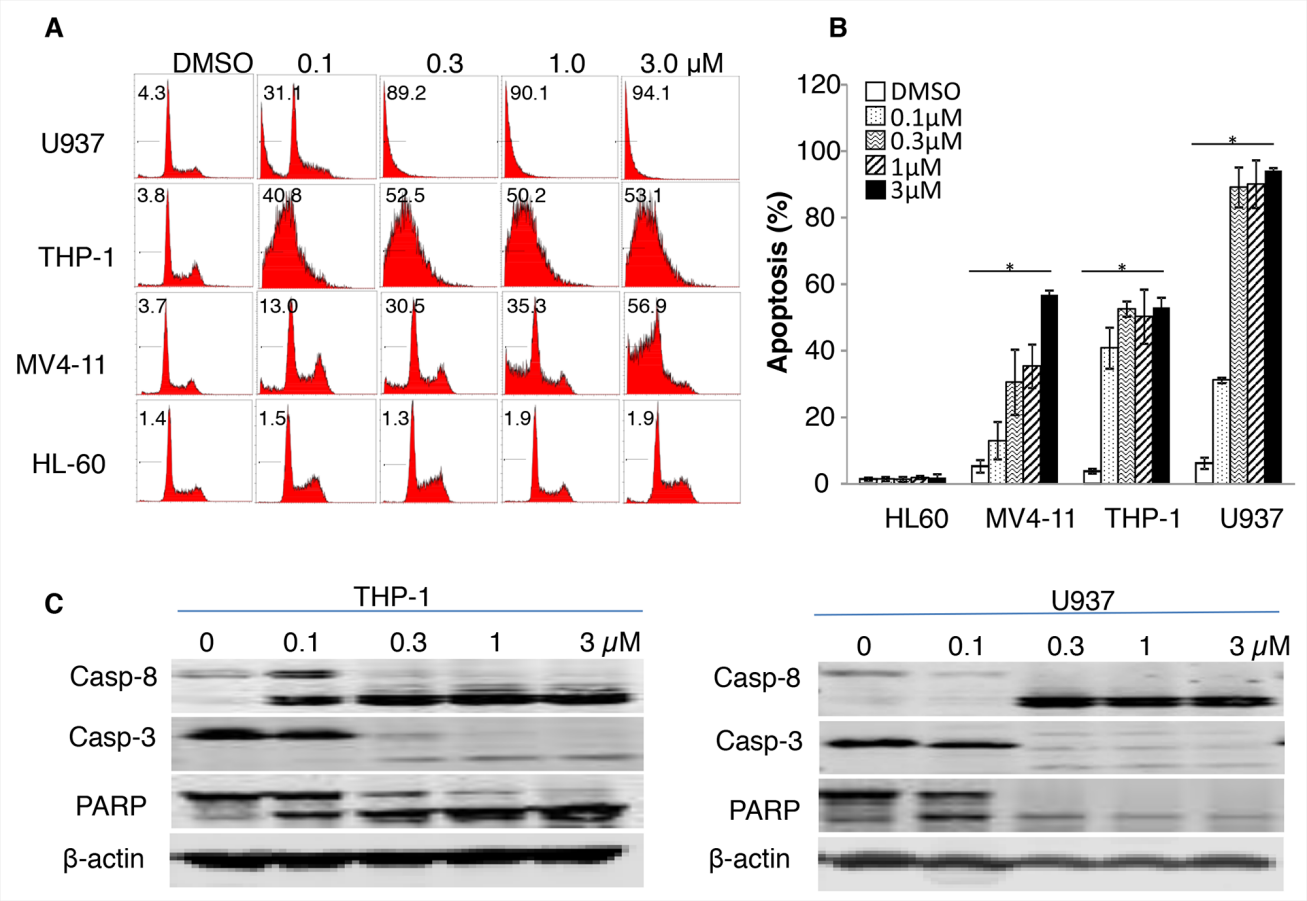
Apoptosis induction by NSC-743380 U937, THP-1, MV4-11, and HL-60 cells were treated with different concentrations of NSC-743380 (0.1 to 3 μM) for 24 h and then harvested and incubated with propidium iodide for 30 min. Apoptosis was detected by flow cytometry analysis. **(A)** The number in each panel indicates the percentage of apoptotic cells. **(B)** Apoptosis percentages are shown, as means ± standard deviations. ^*^ P<0.05 when compared with DMSO treated cells. **(C)** Western blot analysis. THP-1 cells were treated with various concentrations of NSC-743380 as indicated for 24 h. Activation of caspase 3, caspase 8, and PARP were detected by Western blot analysis. β-actin was used as the loading control.

We previously reported that leukemia cells expressing SULT1A1, mostly AML cells, are susceptible to NSC-743380 treatment [18]. In a repeated cell viability assay, we found that treatment of cells from the SULT1A1-expressing AML cell lines U937, THP-1, and MV4-11 with NSC743380 resulted in *in vitro* growth inhibition, with IC_50_ values between 0.1 and 0.8 μM (data not shown). In contrast, SULT1A1-negative HL60 cells were resistant to NSC-743380, with IC_50_ above 10 μM, the highest concentration tested. To determine whether NSC-743380–mediated growth inhibition in these cells is caused by cell cycle arrest or apoptosis, we analyzed cell cycle profiles after the cells were treated with 0.1–3 μM NSC-743380 for 24 h. Cells treated with DMSO were used as controls. In U937, THP-1, and MV4-11 cells (NSC-743380 sensitive), treatment with NSC-743380 led to dose-dependent apoptosis (sub-G1), which is significant when compared with control cells (P < 0.05) (Figure 1). Apoptosis was induced in 53%-94% of THP-1, MV4-11, and U937 cells with 3 μM NSC-743380, whereas the control cells had background level of apoptotic cells (<5%). In contrast, no significant increase in apoptosis occurred in HL-60 cells (NSC-743380 resistant) relative to the control cells. We also performed Western blot analysis to validate the apoptosis induction by NSC-743380. Treatment of THP-1 and U937 cells with NSC-743380 at 0.1 to 3 μM triggered obvious cleavage of caspase 3, caspase 8, and poly(ADP-ribose) polymerase (PARP), demonstrating that NSC-743380 induced robust apoptosis in AML cells.

### NSC-743380 abrogates cFLIP expression in NSC-743380–sensitive leukemia cells

**Figure 2:**
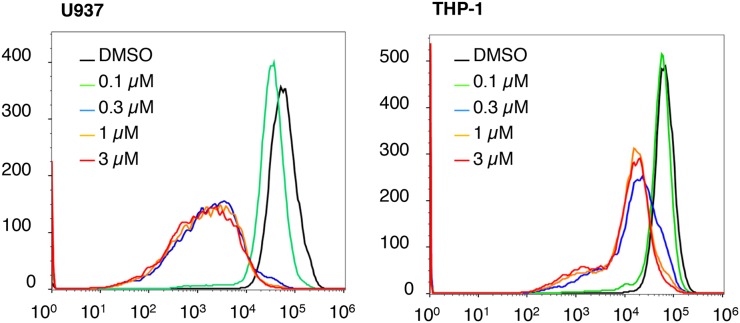
ROS status after NSC-743380 treatment U937 and THP-1 cells were treated with different concentrations of NSC-743380 or DMSO for 6 h. Intracellular ROS levels were determined by flow cytometric assay after cells were stained with H_2_DCF-DA. Treatment of U937 and THP-1 cells with NSC-743380 did not increase but did reduce intracellular ROS levels. Similar results were obtained when the cells were treated with 1 μM NSC-743380 for 30 min to 6 h (data not shown).

**Figure 3:**
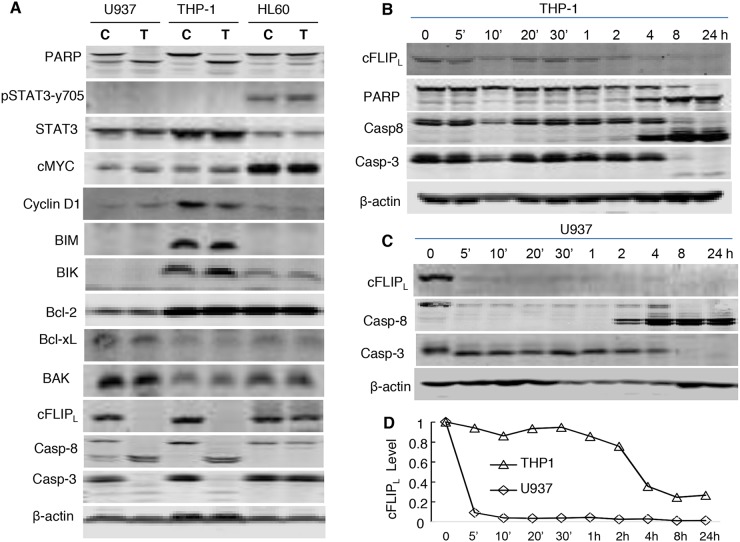
Modulating functions of multiple pathways by NSC-743380 **(A)** Western blot analysis of protein levels. U937, THP-1, and HL-60 were treated with 1 μM NSC-743380 for 72 h. C, controls; T, cells treated with NSC-743380. The levels of each protein indicated were detected using a specific antibody as described in the Methods. **(B, C)** Time-dependent changes in c-FLIP_L_, caspase 8, caspase 3, and PARP. THP-1 and U937 were treated with 1 μM for the indicated times. **(D)** Relative values of cFLIP in (B) and (C) as quantified by ImageJ and normalized with β-actin.

Our previous mechanistic characterization of the anti-cancer activity of NSC-743380 in lung cancer cells revealed that NSC-743380 induced robust ROS and suppressed STAT3 activation, both contributed to the anticancer activity of NSC-743380 and its analogues [11, 12, 16]. We investigated whether similar mechanisms were involved in NSC-743380–mediated apoptosis in AML cells. To this end, we determined whether ROS were induced in AML cells by treatment with NSC-743380. In comparison with cells treated with DMSO, treatment of U937 and THP-1 cells with various concentrations of NSC-743380 for 6 h did not induce any detectable increase in ROS (Figure 2). Similar results were obtained when cells were treated with 1 μM NSC-743380 for various durations (0.5, 2, 4, and 6 h, data not shown), suggesting that ROS induction is not the major mechanism of action of NSC-743380 in these cells. Western blot analysis showed that STAT3 phosphorylation at y705 was not detectable in either DMSO-treated or NSC-743380–treated U937 and THP-1 cells (Figure 3A). There were no obvious changes in cMYC or cyclin D1 upon treatment with NSC-743380, suggesting that STAT3, cMYC, and cyclin D1 are not involved in its mechanism of action.

We then analyzed NSC-743380–induced changes in proteins of apoptosis pathways. No obvious changes were detected in BCL2 family proteins, including BIM, BIK, BAK, Bcl-2, and Bcl-xL, in either the NSC-743380–sensitive cell lines U937 and THP-1 or the NSC-743380–resistant cell line HL60 after treatment with 1 μM NSC-743380. In contrast, the expression of cFLIP was dramatically suppressed after treatment with NSC-743380 in U937 and THP-1 cells, but not in HL60 cells. A time course analysis showed that obvious cFLIP suppression occurred 5 minutes to 4 h after the initiation of NSC-743380 treatment of U937 and THP-1 cells, before caspase 8 and caspase 3 cleavage, suggesting that cFLIP suppression with the associated caspase 8 activation is a mechanism by which NSC-743380 exerts anti-leukemia activity (Figure 3).

### NSC-743380 inhibits the PI3K/AKT/mTOR pathway in leukemia cells

**Figure 4:**
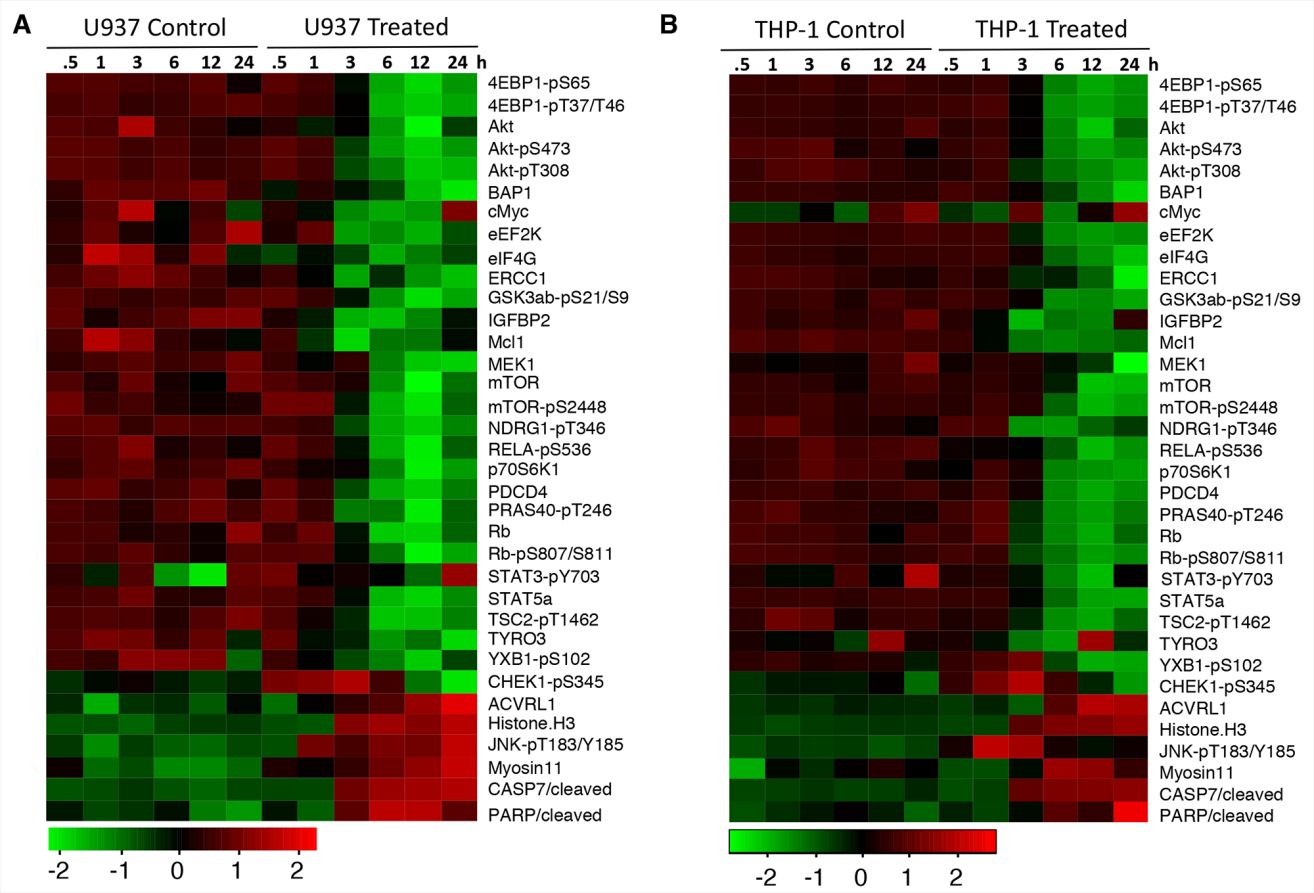
NSC-743380 induced changes in proteins and protein phosphorylation U937 **(A)** and THP1 **(B)** cells were treated with 1 μM NSC-743380 for the indicated times. The cell lysates were subjected to RPPA analysis on 217 protein biomarkers. Heatmaps show the top 30 proteins changed in these two cell lines over time.

To further understand the mechanisms of the anti-leukemia activity of NSC-743380, we analyzed the NSC-743380–induced changes in proteins and protein phosphorylation in U937 and THP-1 cells using a RPPA analysis as we previously reported [14, 19]. Cell lysates were harvested after being treated with DMSO or 1 μM NSC743380 for 0.5 to 24 h and subjected to RPPA analysis for 217 proteins or protein phosphorylation using validated antibodies available in our institution's Functional Proteomics RPPA Core facility. The results showed that treatment with NSC-743380 led to drastic time-dependent suppression of several key nodes in the PI3K/AKT/mTOR pathway and in the protein translation machinery in both U937 and THP-1 cells, including suppression of AKT, eEF2K, eIF4G, and phospho-AKT, -GSK3, -mTOR, -p70S6K, -TSC2, -4EBP, and -PRAS40 (Figure 4). In contrast, histone H3, cleaved caspase 7, cleaved PARP, and phospho-JNK were drastically increased, and phospho-CHEK1 was increased at the early time points but reduced at late time points. We performed Western blot analysis and validated NSC-743380 mediated changes in 4EBP1-pS65, p70S6K and AKT in U937 cells as indicated by the proteomic assay (Supplementary Figure 1). An Ingenuity Pathway Analysis revealed that the PI3K/AKT/mTOR pathway was significantly inhibited at early time points (3 h) (p=2 × 10^-28^). These results strongly indicate that NSC-734480 targets multiple cancer-related pathways and that the PI3K/AKT/mTOR pathway is one of its major targets.

### NSC743380 induces robust apoptosis in primary leukemia specimens expressing SULT1A1

**Figure 5:**
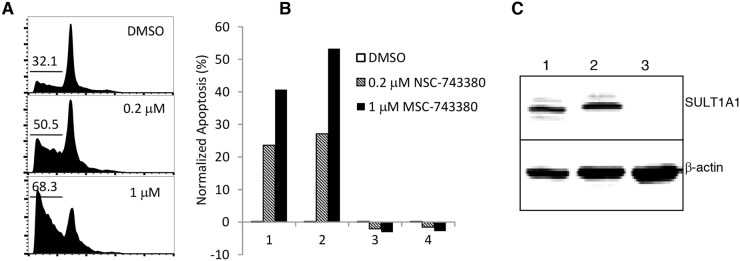
Apoptosis induction in primary AML patient samples (n=4) by NSC-743380 Cells were treated with 0.2 and 1 μM of NSC-743380 for 24 h, while DMSO-treated cells were used as controls. Cells were then harvested and incubated with propidium iodide for 30 min. Apoptosis was detected by flow cytometry analysis. **(A)** An example of fluorescence-activated cell sorter histograms. **(B)** Normalized apoptosis (%) calculated using the equation (apoptosis percentage in treated cells - apoptosis percentage in untreated cells)/(viable percentage of untreated cells) x 100. **(C)** Western blot analysis on expression of SULT1A1 in primary AML.

We investigated whether NSC-743380 can also elicit anti-cancer activity in primary leukemia samples. Mononuclear cells isolated from blood samples from two patients with AML and two patients with chronic lymphocytic leukemia were treated with NSC-743380 at concentrations of 0.2 μM and 1 μM. Cells treated with DMSO served as controls. Percentage of apoptosis was measured by cell cycle analysis or fluorescence-activated cell sorting after staining with annexin V/propidium iodide and was calculated as described in Materials and Methods. Robust apoptosis was induced by the treatment in samples from the two AML patients (samples 1 and 2), but no apoptosis was detected in treated samples from the two patients with chronic lymphocytic leukemia (samples 3 and 4) (Figure 5). A Western blot analysis revealed that SULT1A1 was expressed in the AML samples but not in the chronic lymphocytic leukemia sample that was testable (sample 3; sample 4 was too small for Western blotting) (Figure 5). These results indicate that SULT1A1 expression is detected in primary AML cells and is associated with NSC-743380 sensitivity in primary leukemia samples. Although it is not clear whether the induction of apoptosis by NSC-743380 is specific to AML, the results suggest that NSC-743380 may benefit some AML patients.

### The anti-leukemia activity of NSC743380 is not affected by the presence of normal bone marrow stromal cells

**Figure 6:**
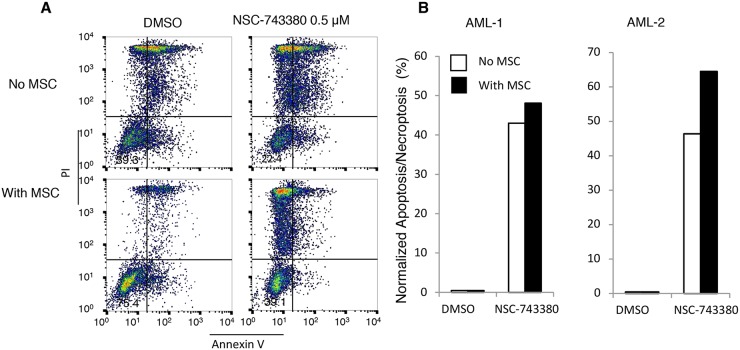
Effect of mesenchymal stem cells on NSC-743380-induced apoptosis Primary AML cells isolated from patients were cultured with or without MSCs and treated with 0.5 μM NSC-743380 for 24 h. Apoptosis was measured by annexin V/propidium iodide staining. **(A)** An example of fluorescence-activated cell sorter histograms. **(B)** Normalized apoptosis percentages in primary AML cells with or without MSC co-culture. Cells treated with DMSO alone as controls.

Tumor microenvironments and mesenchymal stem cells have been reported to provide survival signals to leukemia cells, thereby inducing resistance to anti-leukemia chemotherapy [20, 21]. Therefore, to make the *in vitro* testing system more like the *in vivo* scenario, we tested whether co-culture of leukemia cells with MSCs from normal human bone marrow would have any impact on NSC-743380–induced anti-leukemia activity. Cryopreserved mononuclear cells from the two AML patients were placed into six-well plates with or without MSCs (about 80% confluency) and treated with DMSO or 0.5 μM NSC-743380 for 24 h. Viable cells and apoptotic or necroptotic cells were then quantified by annexin V/propidium iodide staining and normalized to the control cells as described above. The presence of MSCs improved the survival of leukemia cells in culture, but the proportions of apoptotic/necroptotic cells in the treated samples were slightly higher in the presence of MSCs (Figure 6). This result suggested that the anti-cancer activity of NSC-743380 is unaffected by the presence of MSCs.

## DISCUSSION

Our results demonstrated that NSC-743380 induces robust apoptosis and dramatic inhibition of cFLIP expression and of the expression and/or phosphorylation of multiple key nodes in the PI3K/AKT/mTOR pathway in some AML cells, suggesting that NSC-743380 and its analogues can be candidates for AML therapy. Both the cFLIP and the PI3K/AKT/mTOR pathways have been investigated intensively as targets for treatment in various types of cancers, including AML. The PI3K/AKT/mTOR pathway is one of the major signaling pathways that regulate cell growth, proliferation, metabolism, and survival and is among the most commonly deregulated pathways in cancer [22, 23], including in AML [24, 25]. Inhibitors of the PI3K/AKT/mTOR pathway have been shown to elicit anti-leukemia activity in cultured AML cells [24, 26, 27].

Recently, emerging evidence suggested that the anti-apoptotic protein cFLIP is an anti-cancer target. Also known as CASP8 and FADD-like apoptosis regulator (CFLAR), cFLIP is a catalytically inactive homologue of caspase 8 that is essential for inhibiting death receptor–mediated apoptotic and necroptotic cell death [28, 29]. Conditional knockout of cFLIP in hepatocytes [30], intestinal epithelial cells [30, 31], and skin epidermal cells [32, 33] in transgenic mice caused apoptosis in those cells. Overexpression of cFLIP has been reported in a number of primary tumor tissues, including AML [34, 35]. cFLIP overexpression is an independent marker of poor prognosis for most of cancers, suggesting that cFLIP plays a role in cancer progression and resistance to anti-cancer therapy. Indeed, cFLIP overexpression enables tumor cells to escape T cell–dependent immunity *in vivo* [36]. *in vitro* and *in vivo* downregulation of cFLIP with an antisense phosphorothioate oligonucleotide resulted in caspase 8 activation and apoptosis in cancer cells derived from lung, colorectal, and prostate cancer but not in normal cells or in cancer cells sensitized to TRAIL-induced and chemotherapy-induced apoptosis [37]. These findings together with those of our current study indicate that cFLIP is a potential target for anti-cancer therapy in AML. Intriguingly, we did not found NSC-743380 induced cFLIP changes in lung cancer cell H460, which is sensitive to NSC-743380 (data not shown) [11, 12], suggesting that the mechanisms of NSC-743380 mediated apoptosis in AML cells might be different from those observed in lung cancer cells [11, 12].

Little is known about pharmaceutical agents targeting cFLIP in cancer cells, but some therapeutic agents have been shown to downregulate cFLIP, including histone deacetylase inhibitors [38, 39], mTOR inhibitors [40, 41], proteasome inhibitors [42, 43], and a Cox-2 inhibitor [44]. Because the PI3K/AKT/mTOR pathway is reported to regulate cFLIP expression or degradation [41, 45], it is possible that the NSC-743380–mediated inhibitions of cFLIP expression and the activity of the PI3K/AKT/mTOR pathway are connected. However, the time-dependent analysis in the current study suggested that inhibition of cFLIP expression can occur quickly, from 5 minutes to 2 ~ 4 h after the start of treatment with NSC-743380, and is likely independent from the inhibition of PI3K/AKT/mTOR, which was inhibited mostly at 6 h after the start of the treatment.

Our results revealed that NSC-743380 is active against primary AML cells and that its activity is not affected by co-culture with bone marrow MSCs. Bone marrow microenvironments, including bone marrow MSCs, have been reported to play a critical role in drug resistance by protecting cells from the cytotoxic effect of anti-cancer agents [20, 21]. In our *in vitro* study, we found that co-culture of primary AML cells with bone marrow MSCs promoted survival of leukemia cells. Nevertheless, NSC-743380–induced apoptosis in primary AML cells was not reduced by the presence of bone marrow MSCs, suggesting that the anti-leukemia activity of NSC-743380 is not affected by some microenvironment factors that may cause drug resistance for other anti-cancer agents. Moreover, we found that the primary AML samples that were sensitive to NSC-743380 expressed SULT1A1, a biotranformation enzyme, confirming our previous finding that NSC-743380 induces apopotosis in SULT1A1-expressing cells [18].

There are limitations to our study. Only a small number of primary AML samples were tested in our study. It is not clear whether AML samples can be stratified by SULT1A1 expression or whether anti-cancer agents such as tamoxifen, whose activity in breast cancer cells is potentiated by SULT1A1 expression [46], are also active in SULT1A1-expressing AML cells. The primary target of NSC-743380 remains to be identified, and the compound needs further optimization before its use can be translated to clinical applications. Despite these limitations, our results provide proof-of-concept evidence that AML cells expressing SULT1A1 can be targeted by small molecules that induce SULT1A1-dependent apoptosis in malignant cells.

## MATERIALS AND METHODS

### Cell lines and cell culture

The human AML cell lines U937, THP-1, MV4-11, and HL-60 were cultured in Roswell Park Memorial Institute (RPMI) 1640 medium with 10% fetal bovine serum, 100 μg/mL penicillin-streptomycin (all from Invitrogen, Carlsbad, CA). Cell line authentication was performed at our institution's Characterized Cell Line Core using a short tandem repeat DNA fingerprint assay with a PowerPlex 16 HS kit (Promega, Madison, WI). Primary leukemia cells were isolated from the peripheral blood of AML patients (n=4). All patient samples were acquired after informed consent was obtained according to the Declaration of Helsinki under the protocols approved by the Institutional Review Board of The University of Texas MD Anderson Cancer Center. The mesenchymal stromal cells (MSCs) isolated from human bone marrow were obtained from Dr. Michael Andreeff's laboratory (MD Anderson Cancer Center) and were grown in Alpha Minimal Essential Medium supplemented with 20% fetal bovine serum, L-glutamine, and 100 mg/mL penicillin-streptomycin. Cells were maintained in a 37°C incubator with 95% humidity and 5% CO_2_.

### Chemicals and antibodies

NSC-743380 (1-[(3-chlorophenyl) methyl]-1H-indole-3-methanol, C_16_H_14_ClNO, MW 271.7) was synthesized in our laboratory as previously described [10]. This compound had a purity of ≥98%, as was determined by high-performance liquid chromatography-mass spectrometry analysis. The chemical structure was confirmed by nuclear magnetic resonance assay. The 2',7'-dichlorofluorescein diacetate (H_2_DCF-DA) was purchased from Invitrogen Molecular Probes. Antibodies for Western blot analysis were obtained from Cell Signaling Technology (Danvers, MA) except for β-actin, which was obtained from Sigma-Aldrich (St. Louis, MO). Secondary antibodies were purchased from LI-COR Corp (Lincoln, NE).

### Cell viability assay

The cell viabilities of U937, THP-1, MV4-11, and HL-60 were determined using [3-(4,5 dimethylthiazol-2-yl)-5-(3-carboxymethoxyphenyl)-2-(4-sulfophenyl)-2H-tetrazolium (MTS) (Promega), as described [47]. Cells were seeded at a density of 20 × 10^4^cells/mL in 96-well plates. After overnight incubation, the cells were treated for 72 h with NSC-743380 at increasing concentrations (0 to 10 μM). The IC_50_ value, a dose that causes a 50% reduction in surviving cells compared with dimethyl sulfoxide (DMSO)–treated control cells, was calculated by using the software program CurveExpert version 1.3. All experiments were performed at least twice.

### Reactive oxygen species (ROS) analysis

The method of ROS assay was described in a previous publication of ours [16]. The cells were treated either with different concentrations of NSC-743380 for 6 h or with 1 μM NSC-743380 for different time points (0.5, 2, 4, and 6 h). Subsequently, the cell-permeable non-fluorescent compound H_2_DCF-DA (5 μmol/L) was used for measuring intracellular ROS generation. Cells were incubated for 40 min at 37°C and then returned to a pre-warmed growth medium and incubated for 10 min at 37°C. Cells were then harvested and washed with PBS once. The flow cytometry assays were performed at the Flow Cytometry and Cellular Imaging Facility at MD Anderson Cancer Center. All experiments were performed at least twice.

### Apoptosis analysis

Apoptosis of AML cell lines and primary AML cells were analyzed by flow cytometry with propidium iodide staining (BD Biosciences, San Diego, CA) as previously described [9]. In brief, AML cell lines (2 × 10^5^ cells/mL) and primary AML cells (5 × 10^5^ cells/mL) were seeded in six-well dishes and allowed to grow overnight. AML cell lines were treated with 0.1 μM to 3 μM NSC-743380 for 24 h. Primary AML cells were treated with 0.2 μM or 1 μM of NSC-743380 for 24 h. After the treatment, cells were harvested and washed once with PBS and then fixed with 70% ethanol overnight at 4°C. Cell pellets were harvested and resuspended with 200 μl of 50 μg/mL propidium iodide/RNase, then incubated 30 min at 37°C in the dark. Flow cytometry and CellQuest software (BD Biosciences) was used to measure the sub-G0/G1 cellular DNA content.

To determine the effect of MSCs on apoptosis, we seeded U937, THP-1, and primary AML cells alone or in a co-culture with MSCs. MSCs were pre-seeded (5 × 10^3^/cm^2^) in six-well plate and incubated overnight for adherence before the addition of primary AML cells (5 × 10^5^ cells/mL). Cells were treated with 0.5 μM NSC-743380 alone or in a co-culture with MSCs for 24 h and then harvested from suspension. Apoptosis was determined by flow cytometry after double staining with annexin V (BD Biosciences) and propidium iodide following the manufacturer's directions. To normalize with spontaneous apoptosis in cultured primary AML cells, the percentage of apoptotic cells in primary AML cells was calculated using the equation (apoptosis percentage in treated cells - apoptosis percentage in untreated cells)/(viable percentage of untreated cells) x 100 as reported [48]. The flow cytometry assays were performed in the Flow Cytometry and Cellular Imaging Facility at MD Anderson. All experiments were performed at least twice.

### Western blot analysis

Western blot analysis was performed to analyze protein expression after cells were treated with NSC-743380. Briefly, cells were washed with cold PBS, then lysed in Laemmli lysis buffer containing proteinase inhibitor cocktail and phosphatase inhibitor cocktail (Roche Applied Science, Penzberg, Upper Bavaria, Germany). The protein concentration was determined using the Bradford method. Equal amounts of lysate (60 μg) were separated by 10% sodium dodecyl sulfate polyacrylamide gel electrophoresis and were electrophoretically transferred to Hybond-enhanced chemiluminescence membranes (GE Healthcare Life Sciences, Marlborough, MA). Membrane was blocked with blocking buffer (LI-COR) at room temperature for 1 h and then incubated with the primary antibody at 4°C overnight. After being washed with PBS with 0.05% Tween three times, the membrane was incubated with 5% fat milk containing secondary antibody (LI-COR) for 1 h at room temperature. The membrane was washed with PBS with 0.05% Tween again, and protein levels were measured with the Odyssey Infrared Imaging System. β-actin was used as a loading control.

### Reverse-phase protein array (RPPA) assay

The cells treated with NSC-743380 or DMSO for 0.5 to 24h were harvested and lysed in reverse-phase protein microarray (RPPA) lysis buffer (1% Triton X-100, 50 mmol/L 4-(2-hydroxyethyl)-1-piperazineethanesulfonic acid (HEPES) pH 7.4, 150 mmol/L NaCl, 1.5 mmol/L MgCl_2_, 1 mmol/L ethylene glycol-bis(β-aminoetheyl ether)-N, N, N’, N’- tetraacetic acid (EGTA), 100 mmol/L NaF, 10 mmol/L NaPPi, 10% glycerol, 1 mmol/L Na_3_VO_4_, 1 mmol/L phenylmethylsulfonyl fluoride, and 10 μg/mL aprotinin) for 20 min with occasional shaking every 5 min on ice. The cell lysate was then submitted to reverse phase protein array assay at the Functional Proteomics RPPA Core facility at MD Anderson as previously reported [14, 19].

### Statistical analysis

Each experiment was performed at least two times separately, and representative examples are shown for Western blots. Data were reported as means ± standard deviations. Treatment groups were compared using a two-tailed Student t-test, and P < 0.05 was considered statistically significant.

## SUPPLEMENTARY MATERIALS FIGURE


