# Supplementary material for: Anti-leukemia activity of NSC-743380 in SULT1A1-expressing acute myeloid leukemia cells is associated with inhibitions of cFLIP expression and PI3K/AKT/mTOR activities
Source: Oncotarget. 2017 Nov 1;8(60):102150–60. doi: 10.18632/oncotarget.22235 (PMC5731942; doi:10.18632/oncotarget.22235)
Supplement: Supplementary file 1 [file oncotarget-08-102150-s001.pdf]

Anti-leukemia activity of NSC-743380 in SULT1A1-expressing acute myeloid leukemia cells is associated with inhibitions of cFLIP expression and PI3K/AKT/mTOR activities

SUPPLEMENTARY MATERIALS

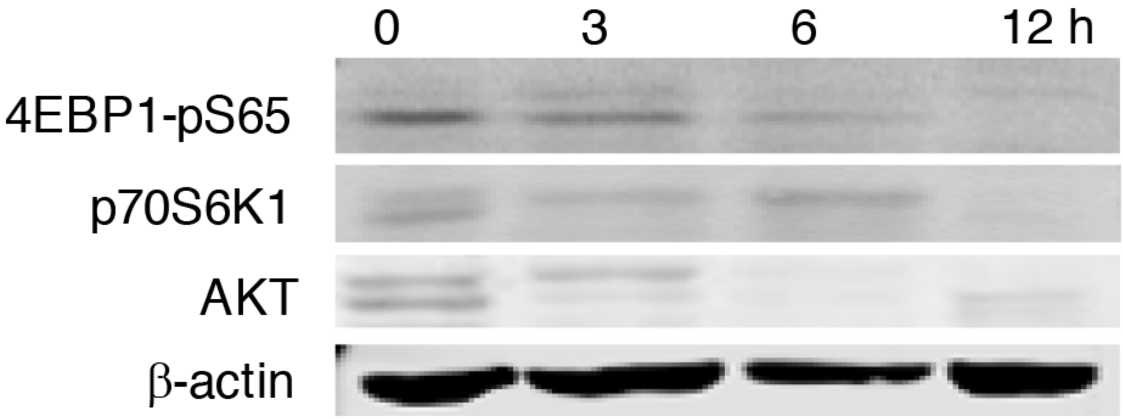

Supplementary Figure 1: Validation of NSC743380 induced protein level changes identified by proteomic assay. U937 cells were treated with 1 µM NSC743380 for the indicated times. Cell lysates were tested for the proteins as indicated. β-actin was used as the loading control.
